# Cadasil - genetic and ultrastructural diagnosis. Case report

**DOI:** 10.1590/1980-57642015DN94000428

**Published:** 2015

**Authors:** Julio Cesar Vasconcelos da Silva, Leila Chimelli, Felipe Kenji Sudo, Eliasz Engelhardt

**Affiliations:** 1 Neuropsychologist; MSc in Internal Medicine/Neurology- x; PhD Student at the Institute of Psychiatry, UFRJ, Rio de Janeiro - Brazil.; 2 Professor at the Department of Pathology - UFRJ, Rio de Janeiro - Brazil.; 3 MD, MSc In Psychiatry - Institute of Psychiatry; PhD Student at the Institute of Psychiatry - UFRJ, Rio de Janeiro - Brazil.; 4 Full Professor (retired) - Federal University of Rio de Janeiro; Cognitive and Behavioral Neurology Unit - Institute of Neurology Deolindo Couto and Center for Alzheimer’s Disease/Institute of Psychiatry - UFRJ, Rio de Janeiro - Brazil.

**Keywords:** CADASIL, skin biopsy, granular osmiophilic material, NOTCH 3, CADASIL, biópsia de pele, material granular osmiofílico, NOTCH 3

## Abstract

Cerebral Autosomal Dominant Arteriopathy with Subcortical Infarcts and Leukoencephalopathy (CADASIL) is a hereditary disorder which affects the cerebral vasculature due to mutations in the NOTCH 3 gene. The diagnosis may be established through genetic testing for detection of these mutations and/or by skin biopsy. We report a case of the disorder in a female patient, who presented recurrent transient ischemic attacks that evolved to progressive subcortical dementia. Neuroimaging disclosed extensive leukoaraiosis and lacunar infarcts. The genetic analysis for NOTCH 3 was confirmatory. The ultrastructural examination of the skin biopsy sample, initially negative, confirmed the presence of characteristic changes (presence of granular osmiophilic material inclusions [GOM]), after the analysis of new sections of the same specimen. The present findings indicate that negative findings on ultrastructural examinations of biopsy should not exclude the diagnosis of the disease and that further analyses of the sample may be necessary to detect the presence of GOM.

## Abstract (pt)

Arteriopatia Cerebral Autossômica Dominante com Infartos Subcorticais e Leucoencefalopatia (CADASIL) é uma desordem hereditária que acomete a vasculatura cerebral devido a mutações no gene do NOTCH 3. O diagnóstico pode ser estabelecido através de testes genéticos para a detecção das mutações e/ou de biópsia de pele. É relatado um caso da desordem em paciente feminino, que apresentou ataques isquêmicos transitórios recorrentes, evoluindo para um quadro progressivo de demência subcortical. A neuroimagem mostrou extensa leucoaraiose e infartos lacunares. A análise genética para NOTCH 3 foi confirmatória. O exame ultraestrutural de biópsia de pele, inicialmente negativa, confirmou a presença de alterações características (presença de material granular osmiofílico [GOM]), após a análise de novos cortes do mesmo material. Os presentes achados indicam que achados negativos de exames ultraestruturais de biópsia não deveriam excluir o diagnóstico da doença e que análise adicional da amostra pode ser necessária para detectar a presença de GOM.

## INTRODUCTION

Cerebral Autosomal Dominant Arteriopathy with Subcortical Infarcts and Leukoencephalopathy (CADASIL) is an early-onset vascular disorder associated with recurrent subcortical transient ischemic attacks (TIA), usually preceded or accompanied by migraine episodes, psychiatric and neurological symptoms and cognitive impairment, and ultimately dementia.^1,2^

Several methods for diagnosing CADASIL have been proposed, including genetic testing, Magnetic Resonance Imaging (MRI) and skin biopsy. The first descriptions of MRI abnormalities in CADASIL date from 1991.^3,4^ Subcortical ischemic lesions (leukoencephalopathy), hypointense on T1 and hyperintense on T2-weighted and FLAIR images, are characteristic findings. In early stages, these lesions are predominantly located in the periventricular regions, centrum semiovale, temporal white matter and external capsule, while lacunar infarcts, are also common early in the disease.^5,6^ Although many patients develop symptoms before 60 years of age, MRI changes may occur before 35 years of age.^4^

Another noteworthy diagnostic method for CADASIL is ultrastructural investigation for granular osmiophilic material (GOM) deposits in the smooth muscle cells of arterioles, obtained through biopsy (skin or muscle). Besides genetic testing for NOTCH 3 and the identification of the NOTCH 3 receptor by immunohistochemistry, ultrastructural analysis of GOM can detect the presence of the disease in cases where the cited methods are not available.

The present article reports a case of CADASIL in which the diagnosis, previously confirmed by genetic testing, was ratified after repeated analyses of a skin biopsy sample, with the aim of: (i) discussing the diagnostic methods; and (ii) highlighting the importance of reiterated analyses of a skin biopsy, even when initially negative, in search of the ultrastructural marker of CADASIL.

## CASE REPORT

A female patient presented with complaints of migraine episodes preceded by visual aura, since the age of 19. At 42, after one such episode, she reported paresthesia and presented hemiplegia. Despite the lack of formal neuropsychological assessment, impairments in language (anomia), memory (forgetfulness for recent events), visuospatial and visuoperceptual abilities (complaints of "distorted vision") were perceived during successive consultations with the psychologist. The cognitive impairments became more severe and progressed to dementia, and the patient died at the age of 57. The Table below depicts the main clinical features presented by the patient, according to medical records.

**Figure 1:**
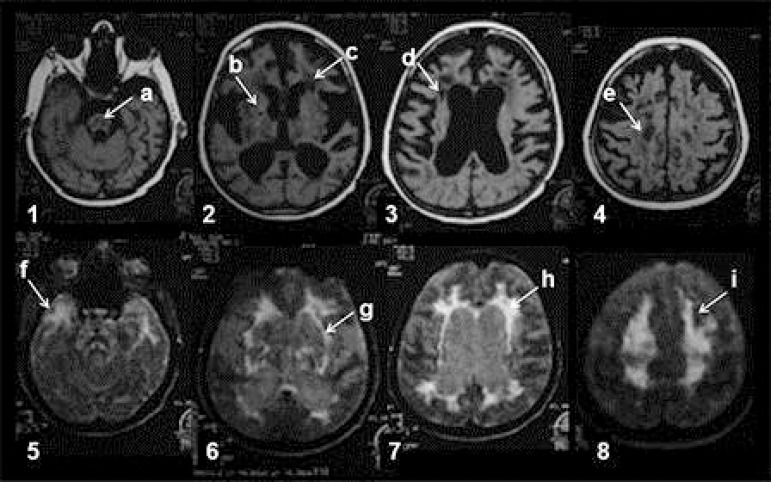
Brain MRI performed at 56 years of age. Upper images [1 to 4] - T1-weighted: [A] brainstem lesion, [B and D] lacunes, [C] whitematter hypointensities, [E] small infarct. Lower images [5 to 6] - FLAIR: [F] hyperintensities in the anterior temporal region, [G] hyperintensity in the external capsule, [H] periventricular hyperintensities, [I] hyperintensities in the centrum semiovale.

Brain MRI at age 56 showed extensive white matter lesions and numerous lacunar infarcts, and dilated supratentorial ventricles (Figure 1).

Initially, a number of different disorders were considered. However, familial history of TIA, migraine and white matter lesions indicated that investigation for CADASIL should be carried out. When the patient was 49, skin biopsy was performed. The sample was analyzed by transmission electron microscopy, which revealed slight thickening of the wall of arterioles; however no GOM bodies were visualized in arteriolar walls. Despite the negative result, the hypothesis of CADASIL was not rejected, and, in 2006, DNA and/or blood samples were sent to France, for genetic analyses,^7^ as detailed in the Box.

**Box:** Description of the genetic analysis performed in the present case.

Samples of DNA and/or blood from the patient and her siblings were sent to the Genetics Laboratory of the Lariboisière Hospital, Paris(*).
The sample of the present case comprised 30 µg of a concentrate containing about 200 ng/µl of DNA. The analysis was performed by direct sequencing of the DNA of exons 3 and 4 of NOTCH 3 (Chromosome 19), which revealed a nucleotide substitution of one arginine (CGC) for one cysteine (TGC) at exon 4 in position 153 (c.535 C>T: R153C).
The examiners concluded that the mutation was typical of CADASIL.
*Department: Genetics of vascular disorders. Principal Investigator: Elisabeth Tournier-Lasserve. Team: Dominique Chiarasini; Anne Joutel; Florence Marchelli; Florence Riant; Manuèle Ruas Benzaken.

**Figure 2:**
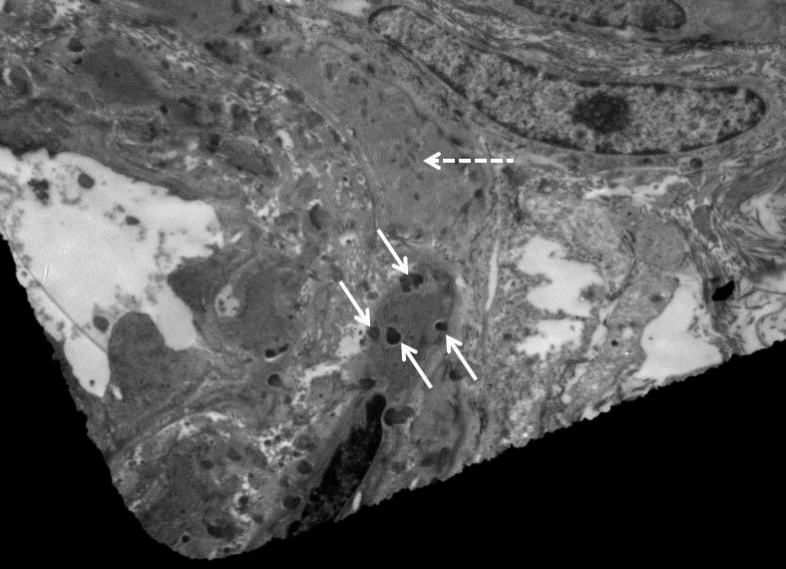
Ultrastructural appearance of skin biopsy, after ultrathin slicing (thickness = 70 nm), post-fixed in osmium, and contrasted with uranyl acetate and lead citrate, showing the presence of GOM in vascular smooth muscle cell. Dashed arrow = vascular smooth muscle cell. Continuous arrows = GOM.

As soon as the results of the genetic analysis were disclosed, the re-examination of the skin biopsy ultrastructure became the focus of renewed interest. The same resin-embedded sample, biopsied eight years before the genetics result, was sliced again, and after due processing, submitted to transmission electron microscopy. Unlike the previous analysis, the repeat analysis detected the presence of GOM (Figure 2).

## DISCUSSION

Past studies have shown that TIA occurs in about two-thirds of individuals with symptomatic CADASIL, commonly followed by cortical-subcortical lesions and disabling manifestations, such as gait disorder, pseudobulbar palsy and cognitive impairment, among others.^8,9^ The mean age for occurrence of these clinical features is around 42 years old,^2^ ranging from 20 to 65 years.^10,11^ The present study describes the presence of these symptoms, similarly to a previous study.^7^

The clinical course was marked by migraine episodes, considered one of the most characteristic symptoms of the disease. A previous study revealed that 22% had migraine with aura.^2^ The first complaints may appear before the age of 20. Hence, these symptoms may occur earlier than the mean age in which brain lesions are evident on MRI images.^12^ In line with a previous study, these symptoms were also evident in the present case.^7^

Additionally, as suggested by Markus et al.,^5^ white matter changes in the anterior temporal pole and in the external capsule can serve as a useful neuroimaging marker for the diagnosis, and moderate to severe changes in the anterior temporal pole may have sensitivity of 89% and specificity of 86% for CADASIL, whereas lesions in the external capsule show high sensitivity (93%) but low specificity (45%). MRI images in this case (akin to a previously published article^7^) depicted lesions in these regions.

Some pathophysiological and diagnostic aspects warrant further consideration. CADASIL is characterized histopathologically by the presence of GOM deposits within small and medium sized cerebral arteries. These inclusions comprise dense deposits with a granular aspect, ranging from 0.2 to 0.8 nm in size. It has been hypothesized that transendothelial transport in CADASIL might be impaired, which may disturb the integrity of the vascular smooth muscle, possibly inducing the appearance of GOM.^13^

As observed in other systemic arteriopathies, vascular changes are present outside the nervous system. For instance, GOM can be found in small vessels of the skin (85% of cases),^14^ muscles (86%),^15^ retina (87%),^16^ and coronary arteries (41% of cases).^17^ The most severe presentation of the disease is brain involvement, in which vascular lesions are characteristically located in the deep white matter and in the basal ganglia.^15-18^

To the best of our knowledge, no previous detailed study has addressed the sensitivity and specificity of biopsy examinations in samples with CADASIL. In some papers, granular material was identified in all symptomatic cases of CADASIL.^19,20^ However, the possibility of negative or false-negative results should not be overlooked.^21^ Ultrastructural analysis results may be influenced by several factors such as choice of contrasting methods, cuts in specific areas, sample storage, among others. Studies have suggested that when using electron microscopy analysis for skin biopsy in cases of suspected CADASIL, special attention should be paid to the quality of the skin sample and to the chosen technique. Although the presence of GOM in veins may be accepted, arterioles (with mean diameter of 20-40 µm) from the deep dermis or superior subcutaneous tissues have been shown to constitute the most suitable samples for the analysis;^22^ thus fragments containing arteriolar segments should be preferred. Due to these limitations, several studies have shown conflicting results regarding the sensitivity of skin biopsy for detecting GOM in patients with genetically confirmed CADASIL.^5,22,23^

A study performed by Joutel et al. (2001)^14^ proposed that immunohistochemical reactions in skin biopsy samples using a specific monoclonal antibody for NOTCH 3 could improve the accuracy of the test for diagnosing the disease. To test this hypothesis, the authors compared the sensitivity and specificity of the method in two groups of patients with suspected CADASIL. Based on the findings, the study revealed that contrasting immune techniques were highly sensitive (96%) and specific (100%) for the diagnosis of CADASIL.

Molecular analysis in the present case, as previously mentioned, revealed a nucleotide substitution of one arginine (CGC) for one cysteine (TGC) in exon 4 at position 153 (c.535 C>T: R153C).

Mutations can occur in various regions of the NOTCH 3 gene and consequently a thorough molecular assessment could be both time consuming and costly. Markus et al.^5^ found 15 mutations in different regions of NOTCH3 in 48 families, 73% of which were in exon 4, 8% in exon 3 and 6% in each one of the exons 5 and 6. Based on this pattern, the authors suggested that the protocol for genetic analysis should follow the screening of these exons. The researchers also underlined the fact that skin biopsy tests may present false-negative results. Peters et al. (2005) identified 54 different mutations in the NOTCH 3 gene in 120 (96%) out of 125 biopsy-positive patients.^23^ In these cases, 58.3% of mutations were located in exon 4 and 85.8% in exons 2 to 6. No mutation was identified in 5 (4%) patients, indicating that false-negative results in genetic testing may also occur. The researchers suggested that highly suspected cases of CADASIL should be submitted to skin biopsy, even if the genetic test shows negative results. In such cases, immunohistochemistry for NOTCH 3 or ultrastructural examination for the detection of GOM could be considered in order to confirm the diagnosis.

In conclusion, the identification of mutations in the NOTCH 3 gene is of indisputable value for the diagnosis of CADASIL, due to its high specificity. Ultrastructural examination of skin biopsies for GOM detection may require a thorough analysis of numerous cuts of a technically adequate biopsy. This procedure might be especially useful to avoid false-negative results in cases where clinical and neuroimaging data strongly support the diagnosis, and when genetic testing is unavailable or yields a negative result.

## Figures and Tables

**Table 1:** Clinical aspects throughout the patient's life.

Clinical features	Age (years)
Migraine	19
Paresthesia in lower left limb	42
Paresthesia spread to the upper left limb, and to the right limbs; left-side hemiparesis; dysphagia; vertigo; cognitive impairment (aphasia [transient]; dysnomia; forgetfulness for recent events, “distorted vision”)	44
Mood disorders (depressive symptoms, anxiety)	45
Right-sided hemiparesis	46
Pseudobulbar palsy; aphasia	53
Dementia and death	57
